# Cortical bone adaptation response is region specific, but not peak load dependent: insights from $\mu$CT image analysis and mechanostat simulations of the mouse tibia loading model

**DOI:** 10.1007/s10237-023-01775-6

**Published:** 2023-10-18

**Authors:** Corey J. Miller, Edmund Pickering, Saulo Martelli, Enrico Dall’Ara, Peter Delisser, Peter Pivonka

**Affiliations:** 1 School of Mechanical, Medical and Process Engineering, Queensland University of Technology (QUT), Brisbane, Australia; 2 Department of Oncology and Metabolism and Insigneo Institute for In Silico Medicine, University of Sheffield, Sheffield, UK; 3 Veterinary Specialist Services, Brisbane, Australia

**Keywords:** Bone adaptation, Region specific, Mechanostat, Mouse tibia loading, Sciatic neurectomy, Simulations

## Abstract

The two major aims of the present study were: (i) quantify localised cortical bone adaptation at the surface level using contralateral endpoint imaging data and image analysis techniques, and (ii) investigate whether cortical bone adaptation responses are universal or region specific and dependent on the respective peak load. For this purpose, we re-analyse previously published $\mu$CT data of the mouse tibia loading model that investigated bone adaptation in response to sciatic neurectomy and various peak load magnitudes (*F *= 0, 2, 4, 6, 8, 10, 12 N). A beam theory-based approach was developed to simulate cortical bone adaptation in different sections of the tibia, using longitudinal strains as the adaptive stimuli. We developed four mechanostat models: universal, surface-based, strain directional-based, and combined surface and strain direction-based. Rates of bone adaptation in these mechanostat models were computed using an optimisation procedure (131,606 total simulations), performed on a single load case (*F* = 10 N). Subsequently, the models were validated against the remaining six peak loads. Our findings indicate that local bone adaptation responses are quasi-linear and bone region specific. The mechanostat model which accounted for differences in endosteal and periosteal regions and strain directions (i.e. tensile versus compressive) produced the lowest root mean squared error between simulated and experimental data for all loads, with a combined prediction accuracy of 76.6, 55.0 and 80.7% for periosteal, endosteal, and cortical thickness measurements (in the midshaft of the tibia). The largest root mean squared errors were observed in the transitional loads, i.e. *F* = 2 to 6 N, where inter-animal variability was highest. Finally, while endpoint imaging studies provide great insights into organ level bone adaptation responses, the between animal and loaded versus control limb variability make simulations of local surface-based adaptation responses challenging.

**Supplementary Information:**

The online version contains supplementary material available at 10.1007/s10237-023-01775-6.

## Introduction

The mouse tibia compression loading model is commonly used to assess bone adaptation due to mechanical loading (De Souza et al. 2005; Sugiyama et al. 2008, 2010, 2012; Birkhold et al. 2014, 2016; Roberts et al. 2020; Nepal et al. 2023). This model has helped researcher understanding of various parameters and relationships in Frost’s mechanostat model, such as range of habitual strains and rates of bone formation and resorption, which have been crucial in developing computational models of bone adaptation (Pereira et al. 2015; Cheong et al. 2020; Oliviero et al. 2018). However, these parameters are often obtained from studies that consider a single peak load.

In the mid-2000s, Skerry argued that a universal (i.e. same for all bone types) mechanostat theory may be an oversimplification of the adaptive processes occurring in bone, but that the latter depend on a number of factors including bone type (e.g. tibia vs femur), spatial position within a certain bone (e.g. proximal vs distal region in the tibia) and the surface they occur on (e.g. endosteal versus periosteal) (Skerry 2006). Skerry argued that the mechanostat may respond differently even within the same bone, providing the example that excessive mass in the distal portion of a limb (e.g. femur, tibia) would reduce locomotion capability; and therefore, a uniformly reinforced bone is not the optimal shape for daily load-bearing activities (Skerry 2008).

Skerry’s arguments are supported by many findings from the mouse tibia loading model; several key supporting observations from these studies include: i) changes in cortical bone area (*Ct*.*Ar*) and cortical thickness (*Ct*.*Th*) depend linearly on applied peak mechanical load (De Souza et al. 2005; Sugiyama et al. 2012; Miller et al. 2021; Ellman et al. 2013; Kotha et al. 2004), ii) adaptation varies along the proximal-distal length of the tibia (De Souza et al. 2005; Sugiyama et al. 2012; Roberts et al. 2020; Miller et al. 2021; Galea et al. 2020; Monzem et al. 2021), iii) *Ct*.*Th* changes within a given cross section of bone are non-uniform around endosteal and periosteal regions (Roberts et al. 2020; Pereira et al. 2015; Miller et al. 2021), iv) the periosteal and endosteal surface experience different amounts of formation and resorption with respect to applied loads (Roberts et al. 2020; Robinson et al. 2021), v) regions experiencing compressive or tensile strains (i.e. approximately corresponding to the posterior and anterior sides of bone) show differing amounts of bone adaptation (Robinson et al. 2021) and vi) the apparent adaptive strain threshold varies between compressive and tensile regions in both cancellous and cortical bone (Yang et al. 2021). These experimental findings support Skerry’s suggestions for the existence of region and site-specific mechanostat models controlling bone adaptation.

Computational bone adaptation models have been developed to simulate and investigate mechano-adaptation in the mouse tibia, using a variety of mechanical signals including: i) fluid flow (Pereira et al. 2015; Carriero et al. 2018; Tiwari et al. 2018), ii) hydrostatic pressure (Scheiner et al. 2016; Pastrama et al. 2018), iii) peak stress/strain (Villette and Phillips 2017; Carpenter and Carter 2008), iv) principal stress/strain (Birkhold et al. 2016; Cheong et al. 2020) and v) strain energy density (Cheong et al. 2020, 2021; Lavaill et al. 2020). Each of these models provide their own strengths and weaknesses regarding representation of the dynamic mechanical signal, algorithmic complexity and implementation, and the ability to predict bone adaptation responses. However, a major challenge in developing region specific models is the computational expense of mechanical signal calculation. Finite element modelling is the current gold standard method of investigating mechanostat parameters (Pereira et al. 2015; Cheong et al. 2020; Oliviero et al. 2018; Carriero et al. 2018). While accurate in calculating mechanical signals, performing calculations across millions of voxel-derived elements is a time-consuming procedure that may take several hours to complete (Pickering et al. 2022). Mechanical beam theory presents itself as an alternative method of performing stress/strain analysis in cortical bone analysis, with solutions obtained in seconds as opposed to hours (Miller et al. 2021; Trichilo 2018; Pickering et al. 2021; Lerebours et al. 2016; Hjelmstad 2007; Bauchau and Craig 2009; Tiwari et al. 2018; Buenzli et al. 2013; Ashrafi et al. 2020; Kumar et al. 2019); this approach has been validated against finite element modelling, proving to be an equivalent strain modelling tool (Pickering et al. 2022).

One further challenge in creating region specific models is the $\mu$CT imaging protocol used in experimental mouse tibia studies. Two types of imaging methods are currently employed to obtain high resolution scans of the tibiae: i) endpoint imaging and ii) longitudinal imaging. Endpoint imaging compares scans of both tibiae at the end of the experiment, comparing the loaded tibia to the unloaded (known as the internal or contralateral control) tibia to determine adaptive bone changes (De Souza et al. 2005; Sugiyama et al. 2012; Birkhold et al. 2016; Pereira et al. 2015; Galea et al. 2020; Robinson et al. 2021; Galea et al. 2015; Meakin et al. 2015; Halloran et al. 2002; Stadelmann et al. 2011). Endpoint imaging is the more traditional approach, and assumes that the contralateral control is indicative of the loaded limb at the beginning of the study. However, this neglects right versus left limb differences which may impact local bone adaptation measurements. Additionally, such variations have impacts on the registration of images to one another, further increasing the difficulty of tracking the adaptation of a discrete location. Longitudinal imaging uses $\mu$CT scans of the same limb at various time points throughout an experiment, in combination with volumetric image registration, to measure bone’s adaptive response (Roberts et al. 2020; Cheong et al. 2021). This technique can be considered the more accurate method to track discrete bone adaptation responses; however, there have been several concerns regarding the effects of radiation dose on cell behaviour and bone remodelling which require a further optimisation of the longitudinal scanning procedure (Grudzenski et al. 2010; Oliviero et al. 2017, 2019). Furthermore, the image resolution obtainable from endpoint scanners is higher than that possible for the currently available in vivo scanners. While longitudinal imaging does provide a better representation of surface-based changes across multiple time points, the technique is still relatively new and the depth of mechanical loading studies does not yet match available endpoint imaging data.

To this end, a highly cited paper exploring the magnitude of the loading response to progressively increasing peak loads was conducted by Sugiyama et al. (2012). This study performed contralateral endpoint imaging to investigate the adaptive response to 8 different peak loads superposed on a reduced habitual loading model (induced through sciatic neurectomy). In our previous study (Miller et al. 2021), we re-analysed the $\mu$CT data and showed that extracting local bone properties from the contralateral study was possible; however, the study did not track surfaces, instead only calculating relative adaptive quantities, i.e. $\Delta Ct.Th$. The two major aims of the current study are: i) to quantify localised cortical bone adaptation at the bone surface level using contralateral endpoint imaging data and image analysis techniques, and ii) to investigate whether cortical bone adaptation responses are universal or region specific and dependent on the respective peak load. To address these aims, we first develop an image registration algorithm to extract endosteal and periosteal bone surfaces of the mouse tibia; this is performed for both loaded and contralateral control limbs at different cross sections (with a focus on the midshaft), and data are subsequently used to compute cortical surface and cortical thickness changes. Secondly, we develop four beam theory-based mechanostat models of cortical bone adaptation that utilise either universal parameters or take into consideration combinations of region-specific and strain direction dependent values. Mechanostat parameters in our simulation studies are derived from optimisation procedures using a subset of the experimental data. Subsequently, we run predictions with our optimised mechanostat model on the remaining sets of experimental data and compare our in silico results to experimental data at the cortical surface and cortical thickness levels. In doing so, we aim to gain novel insights into the mechano-adaptive response, and to analyse whether or not mechanostat parameters are solely dependent on peak load.

## Methods

**Fig. 1:**
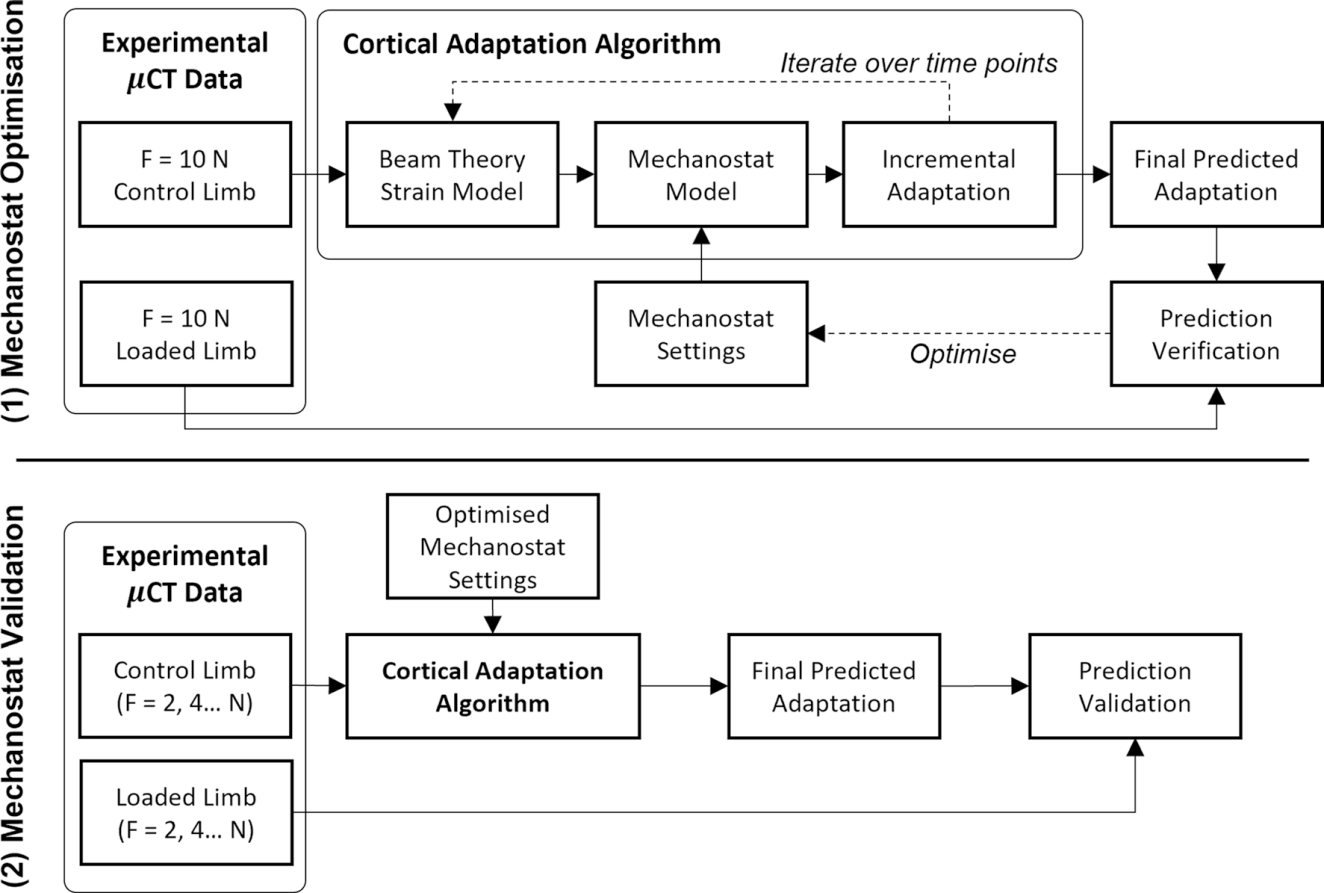
Mechanostat optimisation algorithm and mechanostat validation algorithm flowcharts

The beam theory-based computational model of cortical bone adaptation was developed based on previously published experimental data collected by Sugiyama et al. (2012). The imaging and computational model developments follow the flowchart shown in Fig. 1. Experimental $\mu$CT data were pre- and post-processed, and the local bone adaptive response (i.e. surface propagation and *Ct*.*Th*) was measured. The mean value of $\mu$CT images of the control limb, representing the group population cross section of cortical bone, served as the initial condition for the adaptation algorithm. Mechanical loading was simulated through multiple mechanostat algorithms applied to the cross section in different bone regions. Sciatic neurectomy was simulated by linearly superposing a uniform constant rate of bone loss at the endosteal surface. The multiple mechanostat models were calibrated on a single set of experimental data (i.e. *F* = 10 N) and then validated against data for peak loads of *F* = 0, 2, 4, 6, 8 and 12 N.

### Experimental analysis

This section provides an overview of the methods used for experimental data collection, pre-processing of data, and post-processing of data using image analysis algorithms.

#### Experimental mouse tibia compression model

Experimental data used for this study was previously reported by Sugiyama et al. (2012). A summary of the experimental procedure is provided below, for further details see Sugiyama et al. (2012). 48 mature female C57BL/6 mice were divided into 8 groups (n = 6 mice per group) and were assigned a peak compressive load per group (*F* = 0, 2, 4, 6, 8, 10, 12 or 14 N). For our study, the *F* = 14 N loading case was excluded due to woven bone formation. All mice were subjected to a right sciatic neurectomy at 17 weeks of age (i.e. $t_0$, day 1 of experiment) to induce bone loss due to mechanical disuse. External mechanical loading was applied to the right tibia starting on day 5 and occurring every second day for two weeks. Each loading session consisted of 40 cycles of intermittent loading (trapezoidal wave form, 0.5 N pre-load, 500 N/s loading rate, 0.05 s hold duration, 10 s rest interval). The left tibiae were used as contralateral controls. Mice were euthanised at 20 weeks of age (i.e. $t_{end}$, day 21 of experiment), with both left and right tibiae collected and scanned using $\mu$CT (SkyScan 1172 (SkyScan, Kontich, Belgium), 4.78 $\mu m$ isotropic voxel size).

Sciatic neurectomy has been identified to sensitise the mechanical response of bone to loading (De Souza et al. 2005; Moustafa et al. 2012; Meakin et al. 2015; Delisser 2019; Piet et al. 2019a, b). Mouse tibia adaptation studies that apply loading to an unaltered (i.e. non-neurectomised) tibia utilise loads of between *F* = 10 to 13 N (De Souza et al. 2005; Sugiyama et al. 2008, 2010; Pereira et al. 2015; Piet et al. 2019a); this limited range is due to lower loads inducing no significant adaptive response, whereas higher loads (i.e. *F* >= 14 N) can lead to woven bone formation or fracture of the tibia/fibula. Adding sensitivity to bone formation through sciatic neurectomy therefore enables a deeper exploration of site-specific adaptation and peak load dependency, together with the ability to study the effects of mechanical unloading of the tibia.

#### Image pre-processing

**Fig. 2:**
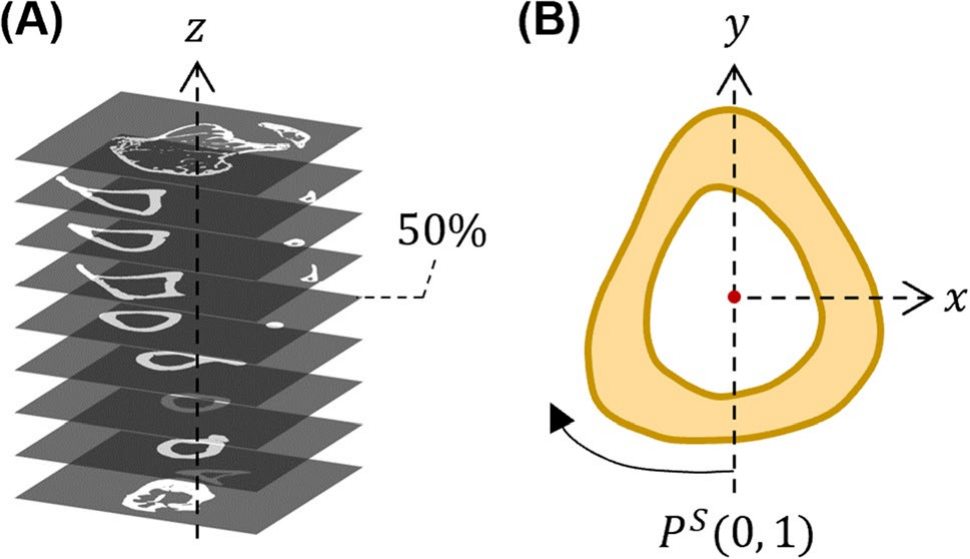
Data alignment and slice selection. **a** Tibial $\mu$CT data are rotated to align such that the long axis of the tibia is aligned with a global z-axis. The z = 50 % cross section is extracted for analysis. **b** Selected cortical bone cross sections are rotated to align minimum $2^{nd}$ moment of area with the y-axis. Point distributions $P^S$ around the cortical surfaces are normalised between 0 and 1 in a clockwise direction, starting at the negative *y* axis

$\mu$CT data were aligned and binarised using MATLAB 2021b as described previously (Pickering et al. 2021). In summary, scans were down sampled by a factor 2 (adjusted isotropic resolution = 9.56 $\mu m$), binarised using Otsu thresholding method, and were rotated such that the principal moment of inertia axis aligned with the z-axis (see Fig. 2 A). All images from the control tibia were flipped horizontally to be able to register the loaded and contralateral cross sections.

The current analyses were performed on the proximal-middle (i.e. z = 37 %) and middle (i.e. z = 50 % cross sections of the mouse tibia, two commonly investigated sections within the limb (Srinivasan et al. 2019; DeLong et al. 2020; Rooney et al. 2022). Unlike in (Sugiyama et al. 2012), who used an average of 100 cortical slices per section for their cortical bone adaptation analyses, we instead use a single cross sectional image; the latter approach has previously been shown to deliver equivalent results describing the adaptation response within a given section (Miller et al. 2021). The methods and results presented here focus on the midshaft, for analysis of the proximal-middle section please refer to Supplementary Fig. 1.

Cross sectional slices were first processed to remove any small cavities (representing blood vessels) that may have been present. The centroid, area and second moments of area were calculated. Images were rotationally registered such that the resulting $I_{min}$ was aligned to the *y* axis and were translationally registered to align the endosteal centroid (i.e. marrow cavity) to a common reference point (Pereira 2014) (Fig. 2). An additional rotation between 1^∘^ to 5^∘^ around the endosteal centroid was manually applied to the control limb cross-sections to better align with the loaded limb for contralateral image analysis. Pixels along the periosteal and endosteal envelopes were identified and mapped into an array; periosteal and endosteal position ($P^P$, $P^E$) distributions were normalised between 0 and 1 in a clockwise direction, with $P^P$, $P^E$ = 0 aligned to the negative *y* axis.

Periosteal and endosteal envelopes of a given cross section are described using the continuous function $\eta$:

1 $$\begin{aligned} \varvec{\eta }^S_{j,i}(t, n, F) \end{aligned}$$

where $\varvec{\eta }^S$ represents the (*x*, *y*) coordinates of a surface point at a given state, *S* indicates the surface (endosteal (*E*) or periosteal (*P*)), *j* represents the selected limb (right loaded limb (*R*) or left control limb (*L*)), *i* represents the index of the surface point, *t* is the time (in days), *n* represents the specimen within the loading group (*n* = 1, 2, 3, 4, 5, 6), and *F* is the applied peak load (i.e. 0, 2, 4, 6, 8, 10 or 12 N).

#### Measurement of local bone adaptation quantities

**Fig. 3:**
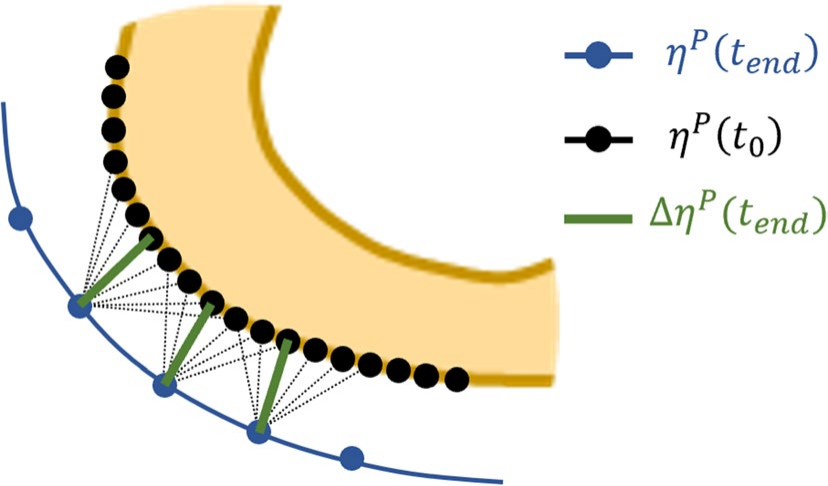
Surface-based adaptation method. The number of points around the unadapted state (i.e. $\varvec{\eta }^S_L$) are interpolated fourfold. Measurements are conducted between each point around the adapted state (i.e. $\varvec{\eta }^S_R$) and all points $\varvec{\eta }^S_L$. The shortest distance ($\Delta \eta ^S$) is selected as the net adaptation distance

For each load case F, local bone adaptation was defined by the difference between the loaded and control tibia. As the mice were considered skeletally mature at commencement of the experiment (i.e. $t_0$ = 1 day), we assume that the control leg stays unaltered from $t_0$ to $t_{end}$, and the initial condition for the loaded leg is the same as the control leg (i.e. $\varvec{\eta }^S_{R,i}(t_0, F) = \varvec{\eta }^S_{L,i}(t_0, F) = \varvec{\eta }^S_{L,i}(t_{end}, F)$, where $t_{end}$ = 21 days)^1^. Surface-based cortical adaptation ($\Delta \eta ^S_i$) at $t_{end}$ can therefore be defined as follows:

2 $$\begin{aligned} \Delta \eta ^S_i = d\left( \varvec{\eta }^S_{R,i}(t_{end}, F), \varvec{\eta }^S_{L,i}(t_{end}, F)\right) \end{aligned}$$

where the function *d* measures perpendicular distance between adapted ($t_{end}$) and baseline ($t_0$) states, evaluated at each cortical point. Net adaptation was calculated by adapting the minimum distance method presented in Miller et al. (2021); the baseline state (i.e. $\varvec{\eta }^S_{L,i}(t_{end}, n, F)$) was interpolated such that the number of points was increased fourfold, the distance between each point $\varvec{\eta }^S_{R,i}(t_{end}, n, F)$) and all interpolated points of $\varvec{\eta }^S_{L,i}(t_{end}, n, F)$ was measured, and the shortest distance was taken as the representative measurement $\Delta \eta ^S_i$ (shown on the periosteal surface in Fig. 3).

Similarly, cortical thickness changes were computed using the hybrid measurement approach reported previously in Miller et al. (2021):

3 $$\begin{aligned} \Delta Ct.Th_i = Ct.Th_{R,i} - Ct.Th_{L,i} \end{aligned}$$

where $Ct.Th_{j,i}$ is the cortical thickness evaluated at $\varvec{\eta }^P_{j,i}$. The hybrid measurement technique calculates thickness using two methods: minimum distance to the endosteal surface, and perpendicular distance to the next cortical edge (periosteal or endosteal). The smaller of the two measurements is taken as the representative thickness for each point, with $\Delta Ct.Th_i$ being the difference between adapted and control limbs evaluated at each point $\varvec{\eta }^P_{j,i}$. This approach is able to manage cortical cross-sections which have an irregular shape, e.g. sections with a pronounced tibial ridge such as the proximal-middle (i.e. z = 37 %) section (see Miller et al. 2021 for further details).

Following all measurements for each mouse *n*, mean and standard deviation values were calculated for $\Delta \eta ^P_i$, $\Delta \eta ^E_i$ and $\Delta Ct.Th_i$. A Student’s t-test was performed within each loading group to identify statistical significance for each of the three measures ($p<$ 0.05 indicates statistical significance). The mean and standard deviation values were used to guide parameter selection within our model; in particular, we note that *F* = 0 and 2 N load cases provide insight into the effects of sciatic neurectomy on cortical bone.

All subsequent analysis and the proposed bone adaptation algorithm will refer to the mean (i.e. population) response of cortical bone, defined as:

4 $$\begin{aligned} \bar{\varvec{\eta }}^S_{j,i} = \bar{\varvec{\eta }}^S_{j,i}(t, F) \end{aligned}$$

For a more compact notation, we will drop the bar from the above equation, with all future reference to $\varvec{\eta }^S_{j,i}$ indicating the mean population value.

### Cortical bone adaptation algorithm

**Fig. 4:**
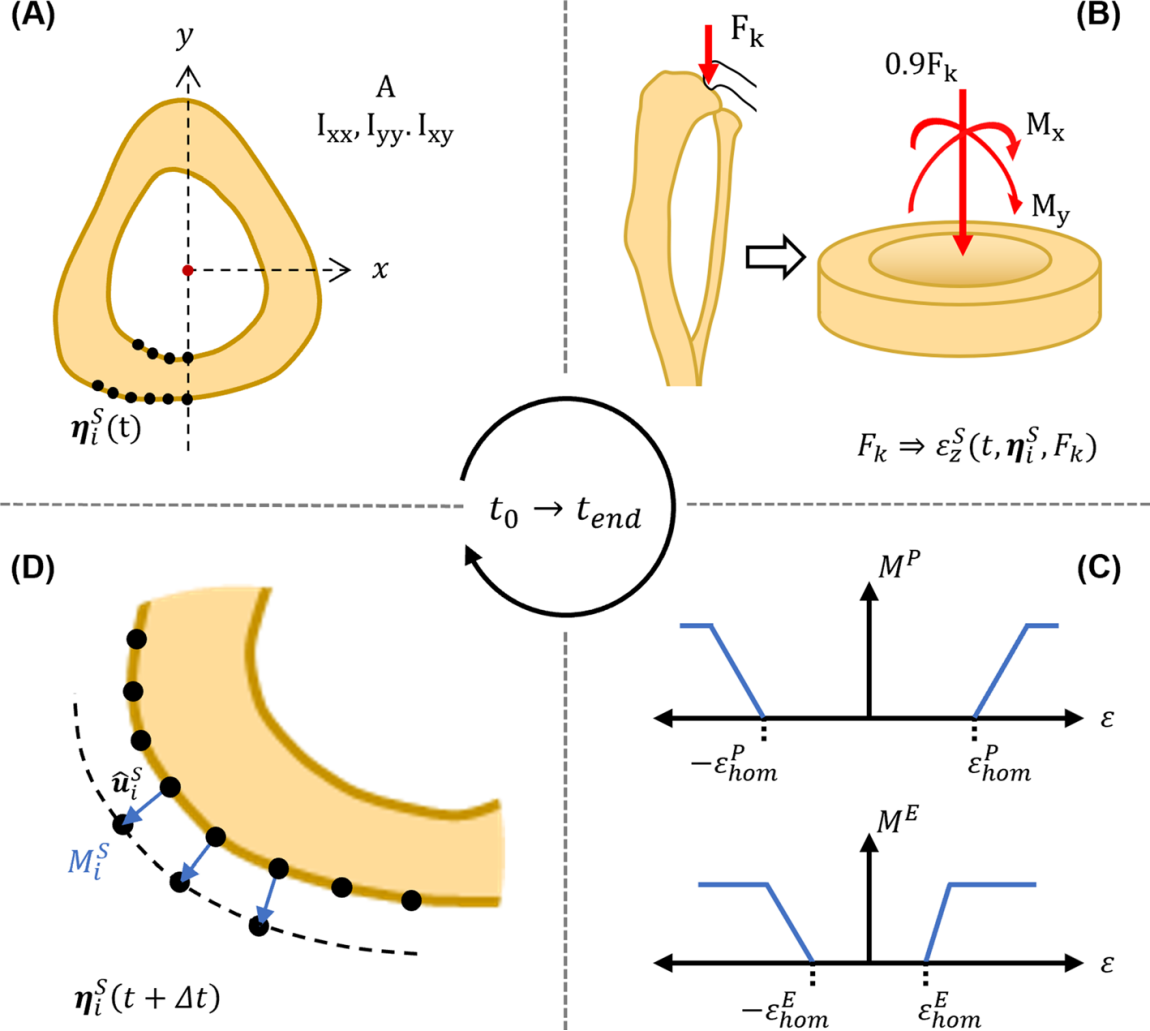
Bone adaptation Algorithm Flowchart. The model follows an iterative, four stage process: (1) Cross sectional properties ($A, I_{xx}, I_{yy}, I_{xy}$) are calculated, (2) externally applied forces *F* are translated into longitudinal strains $\varepsilon ^S_z$ at locations around the cortical shell, (3) strains are compared against a mechanostat model to determine net adaptation *M*, (4) coordinates of all points are updated by the identified net adaptation amount following the normal direction vector $u^S_i$

In the following, we describe the cortical bone adaptation model which is based on a four-step algorithm, outlined in Fig. 4. Using the endosteal/periosteal surface positions at $t_0$ of the loaded leg (i.e. $\varvec{\eta }^S_{R}(t_0, F)$), the algorithm iteratively: 1) extracts geometrical properties of the cross section (centroid, area, second moment of areas), 2) converts peak load into a strain signal using beam theory, 3) runs the mechanostat model, and 4) applies adaptive changes to the endosteal/periosteal surfaces. We use an explicit forward Euler scheme to integrate the mechanostat differential equations together with a step size of $\Delta t$ = 1 day. SN-related catabolism is simulated for 5 days with no additional external loading. The algorithm subsequently simulates 14 days of experimental loading-based adaptation, followed by 2 final days of SN-related bone loss, terminating at $t_{end}$ = 21 days and replicating the experimental procedures. We note that due to registration issues and high standard deviations within the z = 37 % section, reasonable comparisons between simulation and experimental results could not be; as such, the remainder of the methods and subsequent results will relate to the z = 50 % section only.

#### Beam theory for axial strain prediction

A previously validated beam theory approach was used to determine the axial strain in cortical bone (Pickering et al. 2022). For the beam theory approach the ankle was treated as a pin supported joint and an axial compressive load *F* applied at the tibial plateau at position $\varvec{p}_F = (x,y)_F$, replicating the experimental procedure (Pickering et al. 2021). The tibia was assumed to account for 90 % of the peak load applied, with the remaining 10 % attributed to the fibula (Pickering et al. 2021). In addition, the offset loading position induces bending loads (i.e. $\varvec{M} = 0.9 F \times (\varvec{p}_c - \varvec{p}_F)$, where $\varvec{p}_c = (x,y)_C$ represents the cross sectional centroid).

The longitudinal strain at each point $\varvec{\eta }^S_i$ of cortical bone cross section is computed using the generalised flexure formula (Bauchau and Craig 2009):

5 $$\begin{aligned}{} & {} \varepsilon ^S_{z,i}(t, \varvec{\eta }^S_i, F) = \frac{1}{E}\sigma ^S_{z,i}(t, \varvec{\eta }^S_i, F) =\nonumber \\{} & {} \quad \frac{1}{E} \biggl ( \frac{0.9 F}{A} + \left( \frac{M_x I_{yy} + M_y I_{xy}}{I_{xx} I_{yy} - I_{xy}^2} \right) (y^S_i - y_c) \nonumber \\{} & {} \quad - \left( \frac{M_y I_{xx} + M_x I_{xy}}{I_{xx} I_{yy} - I_{xy}^2} \right) (x^S_i - x_c) \biggr ) \end{aligned}$$

where $\varepsilon ^S_{z,i}$ is the longitudinal strain on the surface *S*, *E* is the Young’s Modulus of bone (14.8 GPa, Kohles et al. (1997)), F is the experimentally applied peak load, *A* is the cortical area, $M_x$ and $M_y$ are the bending moments around the *x* and *y* axes, $I_{xx}$, $I_{yy}$ and $I_{xy}$ are the second moments of area of the cross section, $x_i^S$ and $y_i^S$ are the (*x*, *y*) coordinates of the surface point $\varvec{\eta }^S_i$, and $x_c$ and $y_c$ are the (*x*, *y*) coordinates of the centroid.

For the bone adaptation algorithm, we selected longitudinal strain as our effective strain stimulus for cortical bone adaptation. Longitudinal strain has previously been shown to be equally representative of cortical adaptation when compared to alternative measures such as strain energy density (SED) (Cheong et al. 2020). In the mouse tibia loading model, longitudinal strains dominate and hence provide the major contribution to SED compared to shear strains (Trichilo 2018); the values of longitudinal strain also contain directional information (i.e. negative value for compressive and positive values for tensile strains), a property which was required for the current algorithm.

#### Mechanostat model

**Fig. 5:**
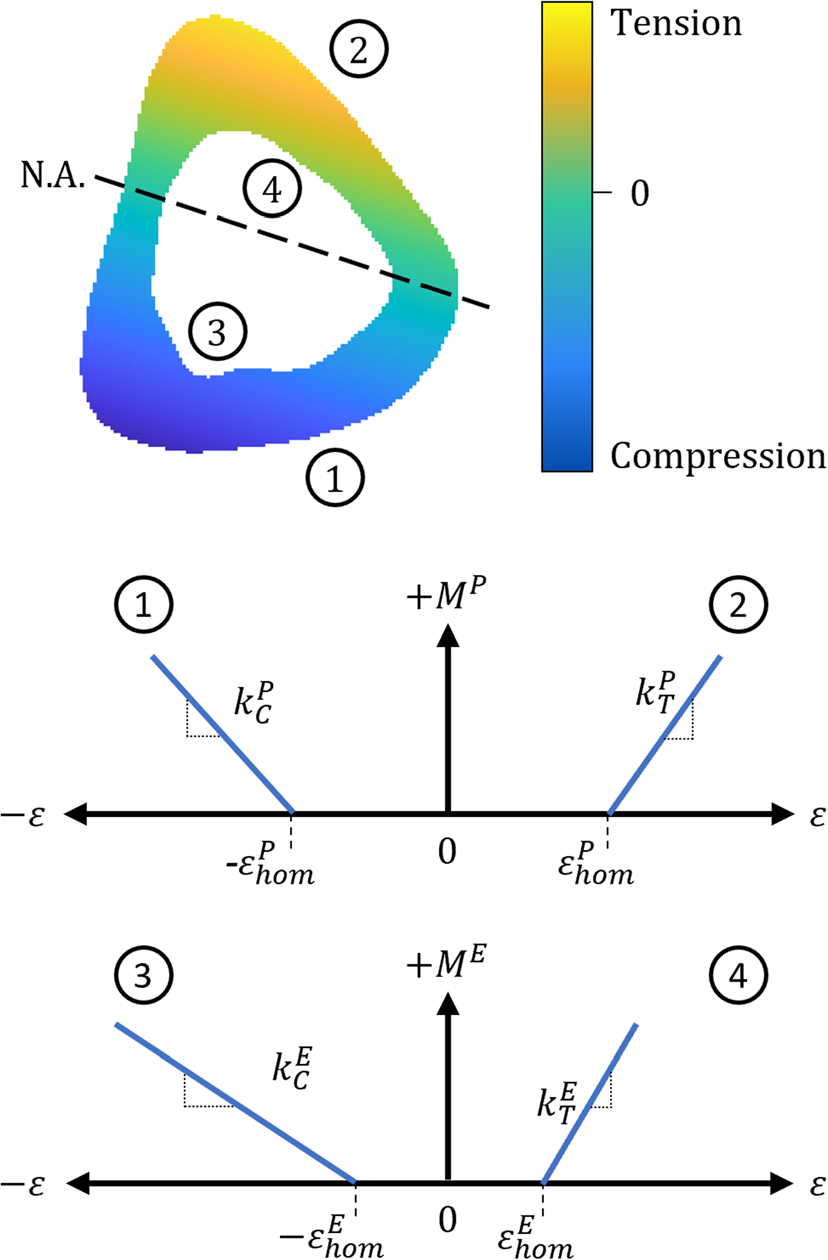
Visualisation of mechanostat relationships for the cortical surfaces. (Top): cortical cross section showing the null axis (N.A.), periosteum under compression (1), periosteum under tension (2), endosteum under compression (3) and endosteum under tension (4). (Bottom): schematic of the mechanostats and their association to cortical surfaces (1), (2), (3), and (4)

As outlined in the introduction, there is evidence that there are different mechanostats in different bone regions (Skerry 2006, 2008; Robinson et al. 2021). We developed four mechanostat models to investigate this: M1: a single, universal formation rate and formation threshold, M2: two formation rates distinguishing between strain directionality (i.e. tension, compression) with a single threshold, M3: two formation rates distinguishing between cortical surfaces (i.e. periosteum, endosteum) with surface-based thresholds, and M4: four formation rates to account for both surfaces in both strain directions with surface-based thresholds (see Fig. 5).

Assuming that bone formation and resorption occurs perpendicular to the periosteal and endosteal surfaces, we can formulate a general scalar evolution algorithm for both surfaces as:

6 $$\begin{aligned} M^S_i = M^S_i(\varepsilon ^S_{z,i}(t, \varvec{\eta }^S_i, F)) \end{aligned}$$

where the scalar incremental surface change ($M_i^S$) is dependent on the time *t* in days, current surface position $\varvec{\eta }^S_i$, and the influence of longitudinal strain $\varepsilon _{z,i}^S$. Using a forward Euler integration scheme, an explicit algorithm to calculate the spatial position of the periosteal and endosteal surfaces can therefore be written as:

7 $$\begin{aligned} \varvec{\eta }^S_{t + \Delta t, i} = \varvec{\eta }^S_{t,i} + M^S_i \Bigr |_{t} \cdot \varvec{{\hat{u}}}^S_i \Bigr |_{t} \cdot \Delta t \end{aligned}$$

where $\Delta t$ indicates the chosen time step, $M_i^S$ represents the net formation calculated through the mechanostat function and evaluated at time *t*, and $\varvec{{\hat{u}}}_i^S$ is the normal unit vector (approximated using coordinates of adjacent points) evaluated at time *t*. During initialisation, the system is assumed to be homeostatic (i.e. $M(t_0)$ = 0), with both surfaces considered as stationary.

The four mechanostat models all follow the same general format. The full mechanostat model can be described using the following equations:

8 $$\begin{aligned}{} & {} M^E_i = {\left\{ \begin{array}{ll} \varepsilon _\text {norm}^E \cdot k^E_C - k^E_\text {SN}, &{} \varepsilon ^E_{z,i}< -\varepsilon ^E_\text {hom} \\ -k^E_\text {SN}, &{} -\varepsilon ^E_\text {hom} \le \varepsilon ^E_{z,i} \le 0 \\ \varepsilon _\text {norm}^E \cdot k^E_T - k^E_\text {SN}, &{} \varepsilon ^E_\text {hom} < \varepsilon ^E_{z,i}\\ -k^E_\text {SN}, &{} 0 \le \varepsilon ^E_{z,i} \le \varepsilon ^E_\text {hom}\\ \end{array}\right. } \end{aligned}$$

9 $$\begin{aligned}{} & {} M^P_i = {\left\{ \begin{array}{ll} \varepsilon _\text {norm}^P \cdot k^P_C, &{} \varepsilon ^P_{z,i}< -\varepsilon ^P_\text {hom} \\ 0, &{} -\varepsilon ^P_\text {hom} \le \varepsilon ^P_{z,i} \le 0 \\ \varepsilon _\text {norm}^P \cdot k^P_T, &{} \varepsilon ^P_\text {hom} < \varepsilon ^P_{z,i}\\ 0 &{} 0 \le \varepsilon ^P_{z,i} \le \varepsilon ^P_\text {hom}\\ \end{array}\right. } \end{aligned}$$

10 $$\begin{aligned}{} & {} \varepsilon ^S_\text {norm} = \frac{\varepsilon ^P_{z,i}(t, \varvec{\eta }^S_i, F) - \varepsilon ^S_\text {hom}}{\varepsilon ^S_{hom}} \end{aligned}$$

where $M_i^S$ is the net adaptation amount ($\mu m/day$), $\varepsilon _\text {norm}$ is the normalised strain difference ($\mu \varepsilon /\mu \varepsilon$), $\varepsilon _\text {hom}^E$ is the homeostatic strain threshold ($\mu \varepsilon$), $k_{sd}^S$ is the rate of adaptation with respect to strain direction *sd* (i.e. tension (*T*) or compression (*C*)) and the normalised strain difference ($\upmu m/day/(\mu \varepsilon / \mu \varepsilon$)$= \mu m/day$), and $k_\text {SN}^E$ is the constant bone resorption rate on the endosteal surface due to sciatic neurectomy ($\mu m/day/(\mu \varepsilon /\mu \varepsilon$)$= \mu m/day$). This resorption term has been included based on our image analysis results (see Sect. 3.1) and with findings in literature, and represents the loss of bone due to the absence of habitual activity, e.g. walking (Sugiyama et al. 2012; Miller et al. 2021; Piet et al. 2019a, b; Kodama et al. 1999). We assume this endosteal resorption is uniform and constant; no loss of bone was observed at the periosteal surface in our image analysis, therefore strains below the threshold elicited no periosteal adaptation response. Strain threshold values were selected based on formation trends identified during experimental data analysis (explained further in Sect. 3.1). Following these observations, we identified that formation thresholds were significantly different between surfaces, but were approximately equal between tensile and compressive regions for a surface. As such, a single formation threshold was selected per surface ($\varepsilon ^E_\text {hom} = 1100 \: \mu$, $\varepsilon ^P_\text {hom} = 2785 \: \mu$). In line with previous studies of mechano-adaptation, our mechanostat model uses a linear relationship between longitudinal strain and the adaptive response (De Souza et al. 2005; Sugiyama et al. 2012; Miller et al. 2021; Schulte et al. 2013; Huiskes et al. 1987; Razi et al. 2015). Adaptation rates $k_{sd}^S$ were calculated per mechanostat model through an optimisation process, discussed in the next section. We use the relative strain change $\varepsilon _\text {norm}^S$ (i.e. the normalised strain difference between observed and threshold strains, calculated in Eq. 10) to better manage the conversion between observed strain, adaptation rates and the net adaptation resulting from the applied load. Comparisons of simulated adaptation at *t* = 21 days and mean experimental adaptation within a loading group were used to evaluate model prediction accuracy. For surface-based evaluation ($\Delta \eta ^P, \: \Delta \eta ^E$), accuracy at a given point $\varvec{\eta }^S_i$ was defined by simulated measurements being within $\pm \: 9.52 \: \mu m$ (i.e. image resolution after down-sampling) of experimental measurements. For $\Delta Ct.Th$, measurement accuracy was defined within a range of $\pm \: 19.12 \: \mu m$ (i.e. two times the image resolution after down-sampling) from experimental measurements. Accuracy results were normalised around the surfaces (i.e. number of accurately simulated points / total number of points) to determine a total surface prediction accuracy.

### Parametric optimisation

The four mechanostat models were optimised using data from the *F* = 10 N load case. Per mechanostat model, formations rates $k_{sd}^S$ used to calculate adaptive changes $M_i^S$ were considered equivalent as follows:

**M1**: Single formation rate ($k^P_T = k^P_C = k^E_T = k^E_C$), single threshold ($\varepsilon ^P_\text {hom} = \varepsilon ^E_\text {hom} = 1100 \: \mu \varepsilon$)

**M2**: Two formation rates ($k^P_T = k^E_T, \; k^P_C = k^E_C$), single threshold ($\varepsilon ^P_\text {hom} = \varepsilon ^E_\text {hom} = 1100 \: \mu \varepsilon$)

**M3**: Two formation rates ($k^P_T = k^P_C, \; k^E_T = k^E_C$), surface thresholds ($\varepsilon ^P_\text {hom} = 2785 \: \mu \varepsilon , \; \varepsilon ^E_\text {hom} = 1100 \: \mu \varepsilon$)

**M4**: Four formation rates ($k^P_T, \; k^P_C, \; k^E_T, \; k^E_C$), surface thresholds ($\varepsilon ^P_\text {hom} = 2785 \: \mu \varepsilon , \; \varepsilon ^E_\text {hom} = 1100 \: \mu \varepsilon$)

Areal properties are subject to change based on the formation occurring around the cortical shell, e.g. changes to the periosteum will influence the strain experienced on the endosteal surface, altering the amount of adaptation that will occur. As such, for all mechanostat models except M1, optimum combinations of formation rates were found in tandem.

Net simulated surface adaptation ($\Delta \eta ^S$) was calculated for both surfaces for a given combination of formation rates. The relative error between simulated (sim) and experimental (exp) results was evaluated using root mean square (RMS) analysis:

11 $$\begin{aligned} \text {RMS}^C = \sqrt{ \frac{1}{i^S_\text {max}} \sum ^{i^S_\text {max}}_{i=1} \left( (\Delta \eta ^S_i)_\text {sim} - (\Delta \eta ^S_i)_\text {sim} \right) ^2 } \end{aligned}$$

where $i_\text {max}^S$ is the total number of cortical points (i.e. 500 per surface). RMS error was obtained for the periosteum (RMS^P^), endosteum (RMS^E^), and combined periosteum and endosteum (RMS^C^). Optimisation for each of the models was conducted in four stages, outlined in Table 1. The first stage, S0, conducted a course sweep of formation rate parameters at an increment of 0.5 $\mu m$. The combination of parameters that provided the smallest RMS^C^ error were selected and used in the next stage of refinement.

**Table 1:** Parameter optimisation stage outlines

Model	Stage	Range	Increment	# of Independent parameters	Total combinations
M1	S0	$0{-}20 \, \mu m$	$0.5 \, \mu m$	1	41
	S1	$S0 \pm 0.5 \, \mu m$	$0.1 \, \mu m$	1	11
	S2	$S1 \pm 0.05 \, \mu m$	$0.01 \, \mu m$	1	11
	S3	$S2 \pm 0.005 \, \mu m$	$0.001 \, \mu m$	1	11
M2	S0	$0 \, \upmu m - 20 \, \mu m$	$0.5 \, \mu m$	2	1681
	S1	$S0 \pm 0.5 \, \mu m$	$0.1 \, \mu m$	2	121
	S2	$S1 \pm 0.05 \, \mu m$	$0.01 \, \mu m$	2	121
	S3	$S2 \pm 0.005 \, \mu m$	$0.001 \, \mu m$	2	121
M3	S0	$0 \, \upmu m - 20 \, \mu m$	$0.5 \, \mu m$	2	1681
	S1	$S0 \pm 0.5 \, \mu m$	$0.1 \, \mu m$	2	121
	S2	$S1 \pm 0.05 \, \mu m$	$0.01 \, \mu m$	2	121
	S3	$S2 \pm 0.005 \, \mu m$	$0.001 \, \mu m$	2	121
M4	S0	$0 \, \upmu m - 8 \, \mu m$	$0.5 \, \mu m$	4	83521
	S1	$S0 \pm 0.5 \, \mu m$	$0.1 \, \mu m$	4	14641
	S2	$S1 \pm 0.05 \, \mu m$	$0.01 \, \mu m$	4	14641
	S3	$S2 \pm 0.005 \, \mu m$	$0.001 \, \mu m$	4	14641

## Results

### Experimental cortical adaptation

**Fig. 6:**
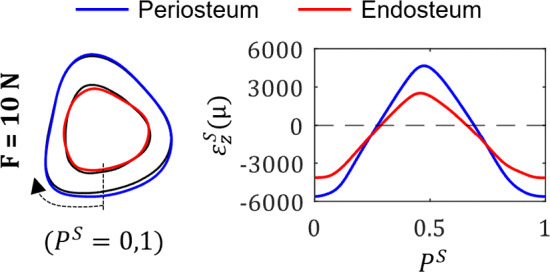
Experimental bone adaptation results of registered mean periosteal and endosteal surfaces (left) and associated longitudinal strain distributions (right) for *F* = 10 N (black line = baseline tibia at $t_0$, blue line = periosteum at $t_{end}$, red line = endosteum at $t_{end}$). Strain profiles calculated on $\eta _R^S(t_{end}, F)$

Experimental results are provided here for the midshaft of the tibia. For results relating to the proximal-middle section, please refer to Supplementary Fig. 1. Figure 6 shows a graphical representation of adaptation (black line = baseline tibia at $t_0$, blue line = periosteum at $t_{end}$, red line = endosteum at $t_{end}$), and the associated strain profile calculated using beam theory in the midshaft of the tibia. For all load cases, peak tensile and compressive strains were observed at approximately $P^S$ = 0.46 and $P^S$ = 0.99 respectively, and the neutral axis lay at approximately $P^S$ = 0.27 and $P^S$ = 0.68.

**Fig. 7:**
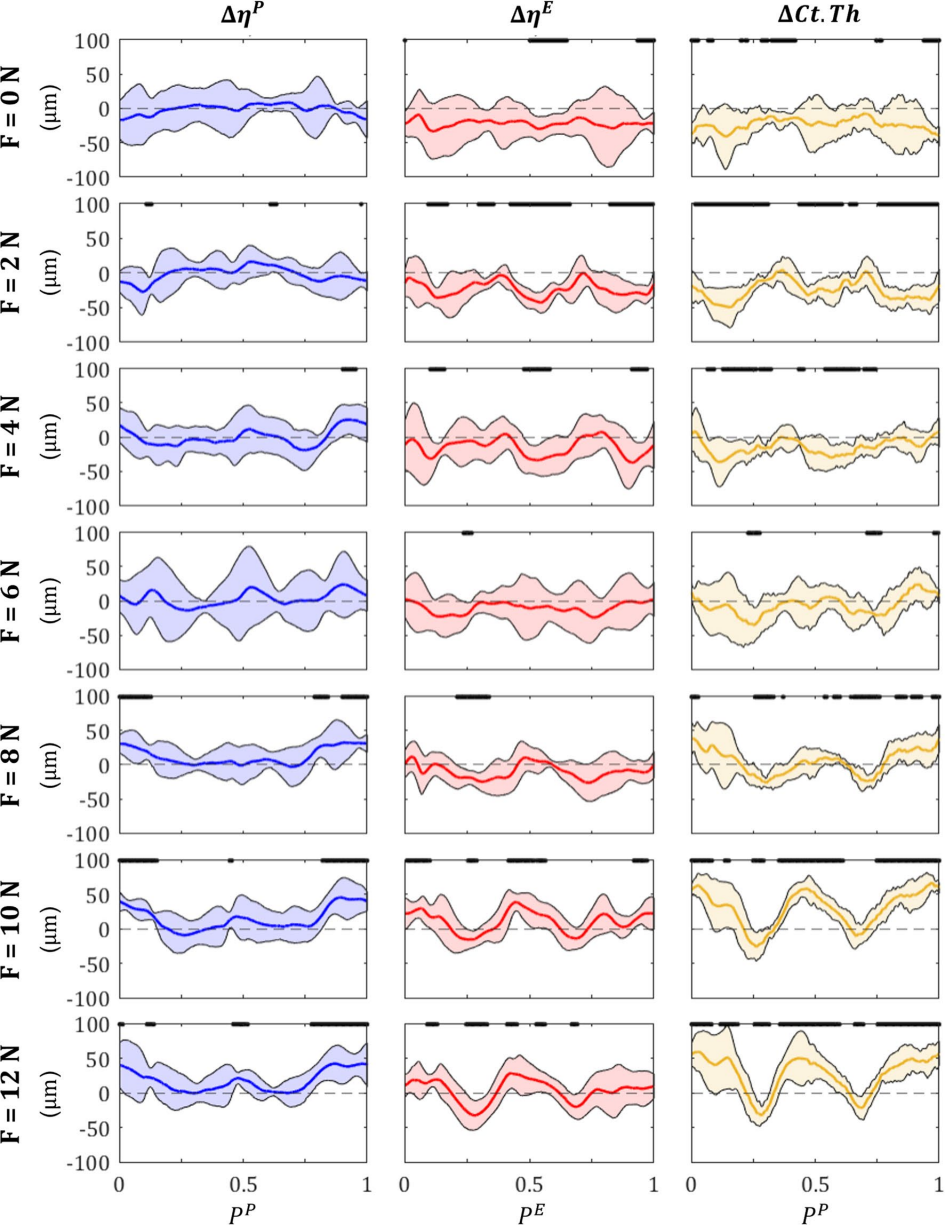
Average experimental changes to the periosteal surface $\Delta \eta ^P$ (*blue*), endosteal surface $\Delta \eta ^E$ (*red*) and cortical thickness $\Delta Ct.Th$ (*yellow*). Solid lines represent the mean adaptive change, shaded areas represent the range of standard deviation, and bold black lines represent surface points $P^S$ that show statistical significance of $p<$ 0.05

Net experimental adaptation measurements are presented in Fig. 7; results of disuse (*F* = 0, 2 N), homeostasis (*F* = 6 N) and overuse (*F* = 10 N) will be highlighted to aid with discussion. Data represented shows the mean (solid line), standard deviation (shaded area) and statistical significance of $p<$ 0.05 (bold black) for $\Delta \eta ^P$ (blue), $\Delta \eta ^E$ (red) and $\Delta Ct.Th$ (yellow). The *F* = 0 N loading case showed no statistically significant growth on the periosteal surface, with the mean $\Delta \eta ^P$ remaining approximately at 0. While the endosteal surface shows minimal statistically significant adaptation, the average $\Delta \eta ^E$ shows a resorption around the entire surface of approximately 21.9 $\mu m$. Similar trends were observed between the *F* = 0 and 2 N loading cases. Here we also observe that the mean $\Delta \eta ^E$ remains approximately 0 for the entire surface. While $\Delta \eta ^E$ does not remain consistent around the endosteal surface as it does for the *F* = 0 N loading case, statistically significant resorption is observed for 55.4 % of the surface, with an average resorption of 22.8 $\mu m$. The endosteal surface change for *F* = 2 N did not follow mechanical loading trends and shared a similar average resorption compared to *F* = 0 N; as such, the endosteal threshold was selected as marginally higher than strain induced via a 2 N load (i.e. $\varepsilon ^E_\text {hom} = 1100 \: \mu \varepsilon$).

The F = 6 N peak load did not induce statistically significant adaptation around the periosteal surface. However, sections of the posterior surface ($0.1< P^P< 0.18, 0.8 < P^P \le 1.0$) show an increase in the mean adaptive response with a peak formation of 23.7 $\mu m$ occurring at $P^P = 0.9$. *F* = 6 N experienced the highest standard deviation across all experimental results, with a range of $\pm \: 63.37 \; \mu m$ occurring at $P^P = 0.5$. The periosteal formation threshold (i.e. $\varepsilon ^P_\text {hom} = 2785 \: \mu \varepsilon$) was calculated as an intermediate strain between *F* = 4 and 6 N, based on the observed formation trends. The endosteal surface experiences an average resorption of 10.1 $\mu m$, with a peak resorption of 24.2 $\mu m$ at $P^E = 0.76$. While the average $\Delta \eta ^E$ shows a trend of resorption, only 3.2 % of this was shown to be statistically significant.

Under the F = 10 N load case, the periosteal surface shows statistically significant formation along the posterior portion ($0 \le P^P< 0.15, \: 0.85 < P^P \le 1.0$) of the cross section, with a peak formation of 45.6 $\mu m$ at $P^P = 0.89$. Several points around $P^P = 0.45$ on the anterior surface of the periosteum show a statistically significant formation of $\Delta \eta ^P \approx$ 14.1 $\mu m$. The endosteal surface shows significant formation events, both on the posterior ($0 \le P^E< 0.1, \: 0.91 < P^E \le 1.0$) and anterior ($0.42< P^E < 0.48$) portions of the surface, with a maximum formation of 38.8 $\mu m$ at $P^E = 0.45$.

### Optimisation of mechanostat parameters

Mechanostat parameters for all four models, along with periosteal, endosteal, and total RMS error, are shown in Table 2. In all models, the endosteal surface experiences a constant resorption of $k_\text {SN}^E$ = $-$1.398 $\mu m$/day, identified through experimental analysis as the average change of the endosteal surface in the *F* = 0 N load case. Formation rates found through the optimisation process differ significantly across each of the four mechanostat models, ranging from a minimum of 0.435 $\mu m$ per normalised strain difference per day (M2, tensile-based formation) to a maximum of 7.055 $\mu m$ per normalised strain difference per day (M4, tension-based formation on the endosteal surface). Of the four models, M4 experienced the smallest error across the periosteal, endosteal, and total surface measurements (RMS^P^ = 6.152 $\mu m$, RMS^P^ = 10.45 $\mu m$ and RMS^P^ = 8.573 $\mu m$, respectively).

**Table 2:** Optimised parameters for mechanostat models

	Units	M1	M2	M3	M4
Homeostatic Strain Thresholds					
$\varepsilon ^P_\text {hom}$	$\mu \varepsilon$	1100	1100	2785	2785
$\varepsilon ^E_\text {hom}$	$\mu \varepsilon$	1100	1100	1100	1100
Sciatic Neurectomy Resorption Rate					
$k^E_\text {SN}$	$\mu m/day$	$-1.398$			
Formation Rates					
$k^P_T$	$\mu m/day$	0.499	0.435	0.986	2.148
$k^P_C$	$\mu m/day$	0.499	0.513	0.986	0.981
$k^E_T$	$\mu m/day$	0.499	0.435	1.056	7.055
$k^E_C$	$\mu m/day$	0.499	0.513	1.056	0.794
Root Mean Squared Error					
RMS^P^	$\mu m$	8.924	8.819	6.481	6.152
RMS^E^	$\mu m$	30.65	30.69	24.53	10.46
RMS^C^	$\mu m$	22.57	22.58	17.94	8.573

In M4, the four rates diverged considerably through the optimisation process; the endosteal surface under tension was the most sensitive to strains, with a formation rate of 7.055 $\mu m$ per normalised strain difference per day. In contrast, the endosteal surface under compression was the least sensitive to strain, with a formation rate of 0.794 $\mu m$ per normalised strain difference per day. Rates on the periosteal surface lie between these two values, with compressive and tensile formation rates of 2.148 and 0.981 $\mu m$ per normalised strain difference per day respectively.

**Fig. 8:**
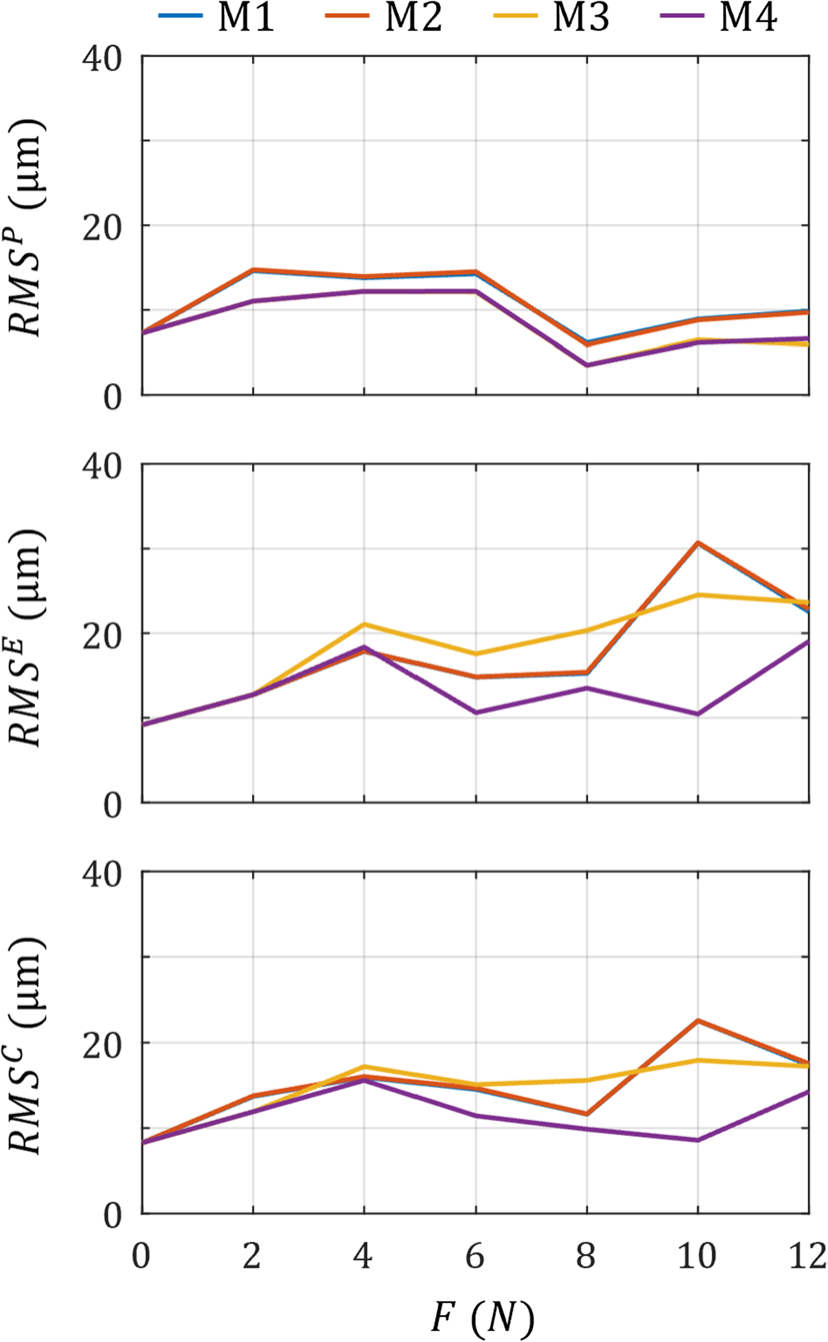
RMS error for the periosteal (top), endosteal (middle) and combined (bottom) surfaces across all loads per mechanostat model (M1 = blue line, M2 = yellow line, M3 = orange line, M4 = purple line). All models were optimised using *F* = 10 N, with parameters used to simulate remaining load cases

All loads were simulated through the optimised parameters above, with associated RMS errors presented in Fig. 8. As no formation-based adaptation was present under the *F* = 0 N load case, RMS error on all surfaces across all models were identical. For RMS^P^, RMS^E^ and RMS^C^, the M1 and M2 models produced a near identical error across all loads. M3 and M4 performed almost equally and provided a lower error than the M1 and M2 models across all loads on the periosteal surface. However, M4 showed distinct improvements over the other models in terms of endosteal surface (RMS^E^) and combined surface (RMS^C^) error measures, with the optimisation load (*F* = 10 N) providing less than half the error of all other models investigated. As such, results in the following section will relate to those obtained from M4.

### Simulated cortical adaptation

**Fig. 9:**
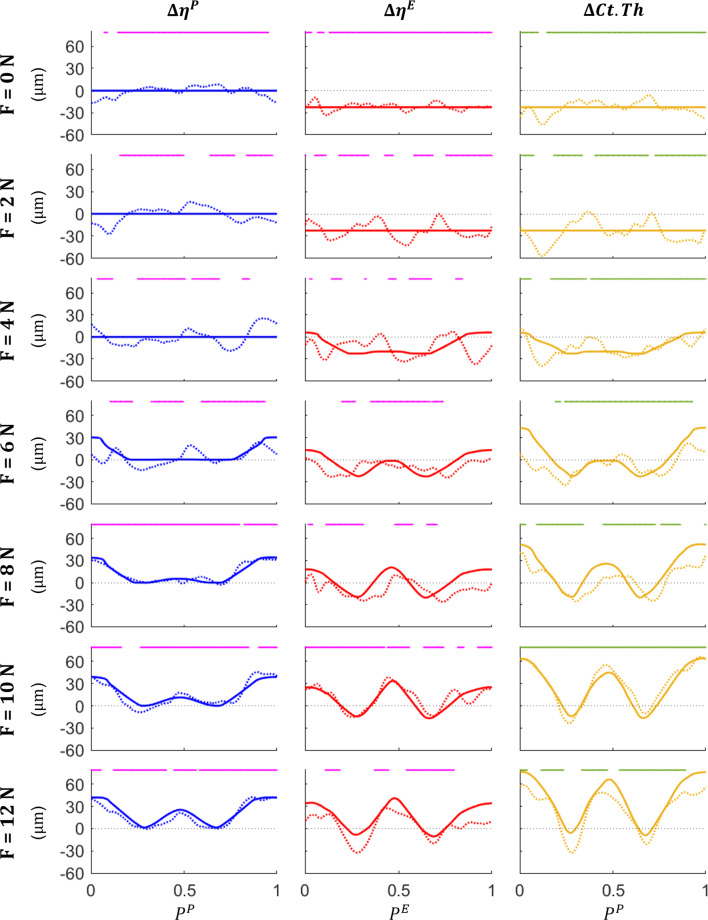
Comparison of simulated (*solid line*) and experimental (*dotted line*) results for bone adaptation at the periosteum (*blue*), endosteum (*red*) and *Ct*.*Th* (*yellow*). Points marked by a magenta line denote simulated measurements within $\pm \: 9.52 \: \mu m$ (i.e. image resolution) of experimental results for the periosteum and endosteum, whereas points marked by a green line denote simulated measurements within $\pm \: 19.12 \: \mu m$ (i.e. two times the image resolution) of experimental results at the *Ct*.*Th* level

**Fig. 10:**
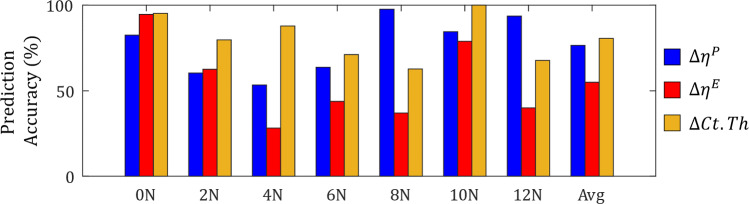
Total surface prediction accuracy for $\Delta \eta ^P$ (*blue*), $\Delta \eta ^E$ (*red*) and $\Delta$ Ct.Th (*yellow*), grouped by peak load magnitude. ’Avg’ prediction accuracy represents the average accuracy across all loads

The difference between predicted and measured adaptation for $\Delta \eta ^P$ (blue), $\Delta \eta ^E$ (red) and $\Delta Ct.Th$ (yellow) has been presented in Fig. 9 (magenta line denotes surface level accuracy, green line denotes thickness level accuracy), and prediction accuracy across all loads is presented in Fig. 10. Evident in Fig. 9, simulated surface level results were substantially more accurate on the periosteum. Simulation accuracy ranged from 53.4 % (*F* = 4 N) to 97.6 % (*F* = 8 N) on the periosteum, while the endosteum ranged from 28.2 % (*F* = 4 N) to 94.6 % (*F* = 0 N), with an average prediction accuracy across all loads of 76.1 % and 55.3 % for the periosteum and endosteum, respectively.

$\Delta \eta ^P$ accuracy was highest when loads were sufficient to produce formation both under tension and compression, with the *F* = 8, 10 and 12 N load cases seeing an accuracy of 97.6 %, 84.6 % and 88.6 %. In contrast, biological noise (expressed as standard deviation) present in the experimental analysis of $\Delta \eta ^E$ was not captured by the simulation, resulting in low prediction accuracies of 38.2 %, 77.0 % and 42.2 % for the same load cases. The *F* = 12 N load case was also observed to experience major resorption around the lateral neutral axis ($P^E \approx 0.36$), experiencing double the amount of resorption when compared to *F* = 10 N (32.40 $\mu m$ compared to 15.36 $\mu m$). *F* = 10 N was also observed to elicit a higher formation amount of 38.82 $\mu m$, compared to 28.47 $\mu m$ in the *F* = 12 N case.

Constant resorption applied to the endosteal surface due to SN was found to accurately predict adaptation for 94.4 % when no additional load was applied (i.e. *F* = 0 N). Due to endosteal geometry fluctuations around the cortical shell, prediction accuracy decreased for *F* = 2, 4, 6, 8 and 12 N. A common region of discrepancy between simulated and experimental results was on the posteromedial edge ($P^E > 0.75$), which the simulation tended to over-predict the anabolic influence of mechanical loading; this phenomenon was less pronounced for *F* = 0, 2 and 10 N.

Accuracy measures from the surface analysis did not directly influence the accuracy of the *Ct*.*Th* measurements. $\Delta Ct.Th$ accuracy ranged from 62.8 % (*F* = 8 N) to 95.2 % (*F* = 0 N), with an average accuracy of 81.29 % across the seven loads. We also note that *F* = 10 N showed 100 % due to being the load case selected for parameter optimisation. $\Delta Ct.Th$ was found to be more accurate than both $\Delta \eta ^P$ and $\Delta \eta ^E$ across all loads with the exception of *F* = 8 and 12 N, where $\Delta \eta ^P$ showed a higher total accuracy. The *F* = 4 N case showed drastic prediction improvement between surface and thickness change measurements, experiencing an accuracy of 53.4 % and a 28.2 % for the periosteal and endosteal surface respectively, but increasing to an 87.8 % $\Delta Ct.Th$ accuracy.

**Fig. 11:**
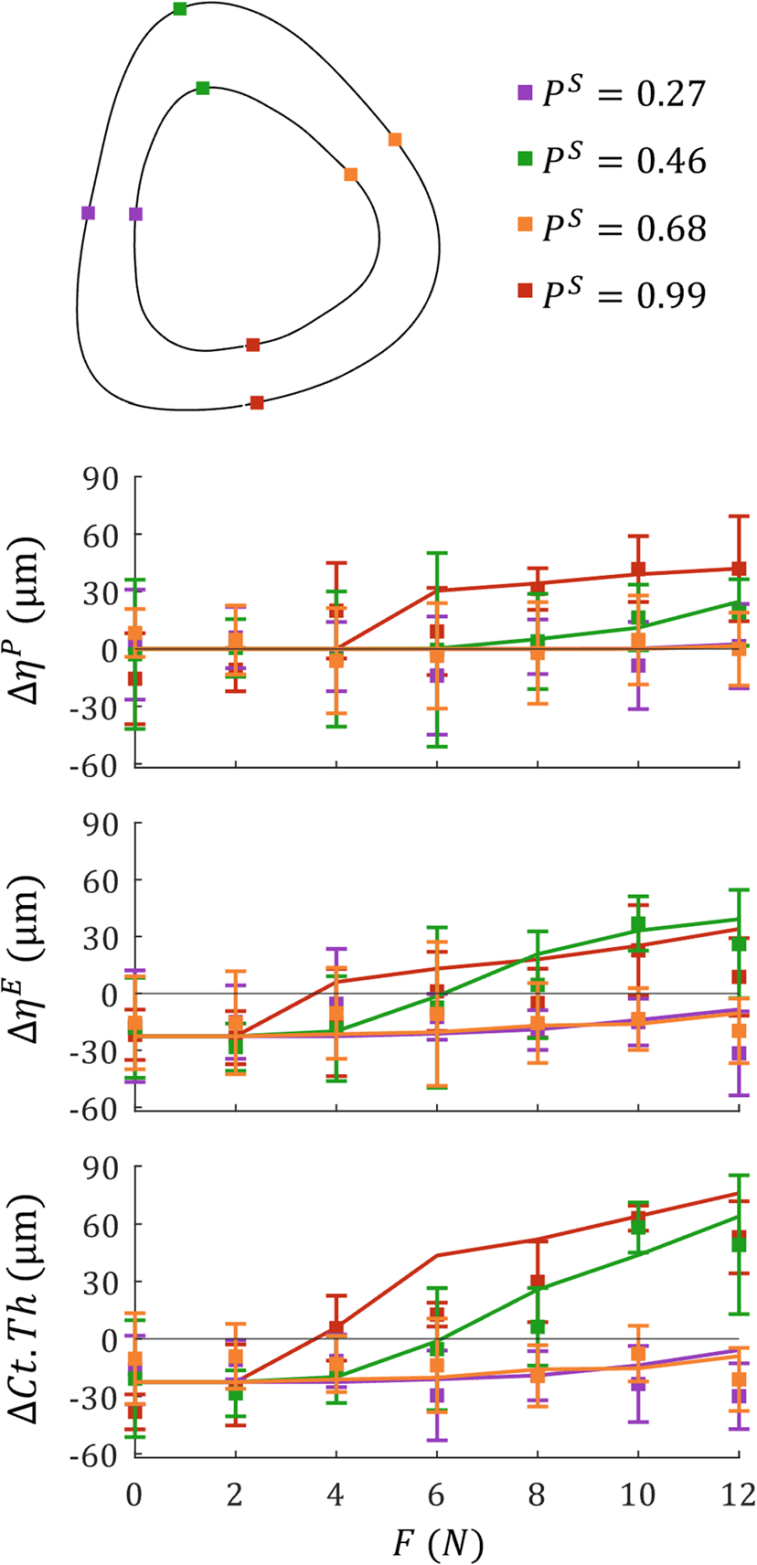
Comparison of net adaption measurements for $\Delta \eta ^P$, $\Delta \eta ^E$ and $\Delta Ct.Th$ across all load cases taken at: peak tension ($P^S = 0.46$, *green*), peak compression ($P^S = 0.99$, *red*), and the neutral axis ($P^S = 0.27$, purple and $P^S = 0.68$, *orange*). Squares with error bars represent the mean ± standard deviation obtained from experimental measurements, and solid lines represent simulated adaptation measurements

Fig. 11 compares simulated net adaption to the mean and standard deviation of experimental adaptation observed in response to each load in four regions around the tibia (0.27 = lateral, 0.46 = anterior, 0.68 = medial, 0.99 = posterior) for $\Delta \eta ^P$, $\Delta \eta ^E$ and $\Delta Ct.Th$. On the periosteum, the posterior region (i.e. compressive region) shows a pseudo-linear formative adaptation response from loads *F* = 6 N and higher, while the anterior region (i.e. tensile region) shows formative events above *F* = 8 N; smaller loads did not induce a formative response. Both the medial and lateral edges (i.e. neutral axis) show approximately no formation or resorption under all loads. Predictions of the simulation fell within the standard deviation for all loads at all locations around the periosteum (Supplementary Fig. 1).

On the endosteum, a constant resorption of approximately 25 $\mu m$ was observed at the medial and lateral edges, as well as on the posterior and anterior regions for loads below *F* = 2 and 4 N, respectively. Loading above this induced pseudo-linear formation, fully countering the effects of SN by *F* = 6 N, and saturating at a net formation of approximately 28 $\mu m$. All values were predicted by the simulation to within standard deviation ranges, except for the posterior region under the *F* = 8 and 12 N load cases which were over-predicted.

The $\Delta Ct.Th$ results approximate to superimposition of $\Delta \eta ^P$ and $\Delta \eta ^E$ per each region analysed, and as such trends are maintained; medial and lateral edges experience a constant resorption of approximately 25 $\mu m$, with pseudo-linear formative trends from loads above *F* = 2 and 4 N on the posterior and anterior regions, respectively. On the $\Delta Ct.Th$ level, the simulation over-represented posterior formation for *F* = 6 N and slightly under-estimated anterior formation for *F* = 10 N; all remaining results falling within standard deviations of the experimental findings.

## Discussion

Our study is the first to investigate the application of multiple mechanostats in a single predictive model of cortical bone adaptation for the mouse tibia loading model. Using a beam theory-based adaptation algorithm, we were able to efficiently calibrate the large number of mechanostat parameters and subsequently perform predictive simulations of bone adaptation on the endosteal and periosteal surfaces. Using a comprehensive experimental data set, our model’s adaptation parameters were validated against several peak load magnitudes. Our results show that mechanostat parameters did not depend on the applied peak loading; rather, the distinction between compressive and tensile strain regions on each cortical surface had a significant influence on the accuracy of the simulation results.

Alignment of $\mu$CT imaging data to extract local bone adaptation properties posed the most significant challenge of the current work. Comparing surface-based changes requires a high degree of precision and is therefore highly sensitive to variations around the cortical shell. Biological variation was likely a major contributing factor; this biological variation can be observed between mice (i.e. different mice have different tibial shapes), but also in the difference between the left and the right leg within a single mouse. The effects of this phenomenon were evident in the high standard deviations and low statistical significance observed, particularly on surface-based experimental analysis (see Fig. 7). This was best exemplified by the $\Delta \eta ^P$ in the *F* = 6 N load case, showing a peak standard deviation of ± 63 $\mu m$ which is over double the maximum formation experienced under the *F* = 10 or 12 N load cases. Additionally, standard deviations observed in the z = 37 % section were considerably higher, with a peak of ± 126 $\mu m$ observed on the endosteum under the *F* = 10 N peak load case. $\Delta Ct.Th$ was less sensitive to alignment issues as it is a relative measurement between surfaces; as long as there is a consistent reference point to align the data to, the orientation of the slice is irrelevant.

However, using information collected from mean surface growth, our model was still capable of achieving high levels of accuracy at predicting mean experimentally observed adaptation, both at the surface and *Ct*.*Th* levels (Fig. 9). Our model presents as a valuable alternative to existing adaptation studies and predictive models that represent cortical adaptation as bone mineral content, bone mineral density or bone volume changes (Robinson et al. 2021; Roberts et al. 2020; Ashrafi et al. 2020; Cheong et al. 2021); by directly modelling geometrical changes, our model is able to capture discrete, surface-based changes that other models are not currently capable of describing.

Our optimisation results show a clear benefit for simulating adaptation using multiple mechanostats. Not only did the optimisation process reveal that multi-mechanostat model M4 produced the smallest RMS^C^ for the optimisation load case (less than half the error compared to M1-M3), but the same parameters also produced the lowest RMS^C^ across all remaining load cases (Fig. 10). The single parameter mechanostat models M1 and M2 were also effective at minimising errors for loads 8N and below, but interestingly provided the worst RMS^C^ for the F = 10 N optimisation load case at over double the error obtained from model M4 (22.57 $\mu m$ and 8.573 $\mu m$, respectively). Simulation models of mechano-adaptation studies are typically optimised around such high loads (e.g. *F* = 10, 12 N) (Pereira et al. 2015; Cheong et al. 2020; Lavaill et al. 2020); our study shows that the single mechanostat model does not provide the best representation for all regions of all surfaces at high loads.

The M4 model shows a clear difference in adaptive rates, both between surfaces and compressive/tensile regions. Shown in Table 2, the endosteal surface under tension (i.e. anterior region) was over 3 times more responsive to strain magnitude compared to the periosteal surface under tension, and was almost nine times more responsive than the endosteal surface under compression. This is likely due in part to two factors: i) the anterior endosteal surface experiences the lowest strain magnitude, and therefore is the last to become mechanically activated, and ii) at high loads (*F* = 10, 12 N), the peak net adaptation in the anterior endosteal region is equivalent to the compressive endosteal and periosteal regions (see Fig. 6). In general, regions of tensile strain within the mouse tibia experienced a greater sensitivity to strains, however this did not translate directly to greater adaptation occurring in these regions given the lower tensile strain magnitude (see Fig. 6). Comparing this to the findings shown in Fig. 9, the discrepancy between strain magnitude and mechano-sensitivity on the endosteum is largely nullified due to high-load saturation, with both tensile and compressive regions experiencing equivalent peak adaptation for loads above 6 N. However, peak periosteal adaptation in the tensile region is far less prominent than the compressive, suggesting elevated strains are not enough to reach the saturation limit. The adaptation patterns observed supports findings of other studies which show that the compressive regions of bone experience a greater volume of formation compared to tensile regions (Roberts et al. 2020; Miller et al. 2021; Robinson et al. 2021), which we identify is due to a reduced response on the anterior periosteal surface.

Our implementation of a resorption due to SN with superposed, beam theory-informed formation proved to be effective method of representing cortical bone adaptation. Assuming a constant, uniform, endosteal resorption due to SN was able to represent SN-induced bone loss in a complete mechanical disuse state (i.e. *F* = 0 N, Fig. 9). At higher peak loads (i.e. *F* = 8, 10 and 12 N), the adaptive response shows a two-peak trend representing the regions of high compressive/tensile strain, and the near-zero strains experienced around the null axis; our bending-based adaptive model is therefore able to correctly predict the same trends. However, when looking at the endosteal surface under the same loads, the mean experimental results show regions of resorption or suppressed formation in areas of high strain, particularly in regions of compression ($P^E<$ 0.25, $P^E>$ 0.75). In fact, inconsistencies to the assumption of bending-induced adaptation can be observed across all loads; for example, under a 4 N load, posterior and anterior regions where strain is elevated show resorption, while areas around the null axis remain near zero. This counters the findings from the *F* = 0 N load case, where all regions experience resorption, including the null axis, and higher loads of 8 N and above, where the null axis still experiences resorption. This may be a result of the cellular environments on the cortical surfaces and their respective responses to mechanical stimuli, or alternatively due to lack of control during experimental loading (e.g. repeatable load location precision); however, such analysis was beyond the scope of the current investigation.

Through verification of our model using the *F* = 0 and 10 N peak load magnitude cases, our results validate the assumption of a linear relationship between strain and formative adaptation in the mouse tibia axial loading model. This assumption had previously been investigated on the *Ct*.*Ar* (Sugiyama et al. 2012) and *Ct*.*Th* (Miller et al. 2021) levels and has formed the basis of several predictive models (Pereira et al. 2015; Cheong et al. 2020; Ashrafi et al. 2020; Lavaill et al. 2020). Highlighted in Fig. 11, the simulated adaptation fell within the standard deviation range of the mean experimental results at the periosteal, endosteal and *Ct*.*Th* levels. The notable outlier is the posterior periosteal surface under a 6 N peak load, which over-predicted $\Delta Ct.Th$. As highlighted previously, the *F* = 6 N loading case showed the highest standard deviation of experimental results, with Fig. 11 showing a decrease in formation from the *F* = 4 N load case in the compressive posterior section. This may indicate that the periosteal surface threshold was not selected correctly, however all loads above 6 N were accurately predicted.

While our four-mechanostat model has added additional complexity to current cortical adaptation algorithms, there is yet more complexity that can be added. In our current model, we opted to select one single formation threshold per surface, extrapolated from experimental data. While other models treat the threshold as a parameter to be optimised for, doing so exponentially increases the required number of simulations to obtain a solution; for instance, stage S0 of optimisation for the M2/M3 models (i.e. two variable parameters) required a 41-fold increase in calculation time over M1, whereas the M4 model (i.e. four variable parameters) took over 2000 times the computational duration. We therefore set thresholds manually to reduce the number of variables and therefore optimisation processing time. However, the mechanostat’s adaptation threshold is a concept that is difficult to directly quantify from experimental data. Using an approximate value enabled the thorough investigation of a four-mechanostat configuration model in a time-efficient manner, revealing the benefits that a more comprehensive mechanostat approach can provide in mechano-adaptation simulations.

Our model also represents experimental loads as a single net daily strain stimulus and does not consider any other habitual daily loading. Sciatic neurectomies were performed to the right hind limbs of each mice to completely mitigate mechanical loading, however the mouse may still be applying small amounts of pressure through limping that would contribute to the daily mechanical strain stimulus. In addition, one would assume the three remaining limbs are required to compensate for the loss of normal function due to neurectomy. However, following sciatic neurectomy, weight bearing has been demonstrated to remain unchanged on the contralateral limb (Kingery et al. 2003). In fact, decreases in epiphyseal bone mineral density in control limbs were observed following contralateral sciatic neurectomy, supporting the assumption that the contralateral limb does not experience increased loading. In future studies of mechanical loading-based adaptation, one could consider bilateral sciatic neurectomy followed by adaptation to the habitual disuse state (approximately 35 days required as per Monzem et al. 2021); this would ensure both legs are adapted prior to unilateral loading, isolating the mechano-adaptation response. Alternatively, tail suspension also provides bilateral disuse (Amblard et al. 2003), but is a more stressful method of disuse in rodent studies.

Finally, while we have shown that the four identified regions of the cortical shell adapt differently, connecting these sensitivities to biological processes was beyond the scope of the present study. An investigation performed by Moustafa et al. (2012) showed that performing a sciatic neurectomy in mice increased the percentage of sclerostin-positive osteocytes within the cortical shell of the tibia, and that application of a mechanical load (*F* = 13.5 N) reduced the percentage to levels significantly below the control, i.e. unloaded, non-neurectomised limb. This is furthered by the studies of Piet et al. (2019a), who showed sciatic neurectomy followed by mechanical loading (*F* = 9 N) exhibits both an increase in osteoblast count and a decrease in osteoclast count on the endosteal surface, as well as an increased periosteal mineralised surface. However, histomorphometry was not factored into the loading study analysed in this paper (Sugiyama et al. 2012); as such, we are unable to comment on the distribution or cellular concentrations on either surface and any differences that may be present across the multiple peak loads investigated.

## Conclusion

In this work, we presented an image analysis framework for experimental mouse tibia endpoint imaging studies that allowed registration of two-dimensional cortical cross-sections of loaded and contralateral mouse tibiae; this enabled the identification of mean endosteal and periosteal surfaces before and after loading, as well as the extraction of local cortical thickness measurements. Applying this framework to the previously collected data of Sugiyama et al. (2012), we investigated bone region- and longitudinal strain direction-dependent adaptation responses, and their potential dependence on peak load magnitude, considering four mechanostat models of varying complexity. Our image analysis and numerical simulation studies provided insights into the mechano-adaptation response:

- Mean local cortical thickness estimation can be readily achieved by image co-registration and suitable normal or closest distance between bone surface measurements.
- Estimation of mean endosteal and periosteal surfaces at the commencement of loading and at study endpoint is challenging due to between-animal variation per group and between contralateral limbs.
- Root mean square error for the multi-mechanostat model (M4) was less than half the error observed in the universal mechanostat model (M1).
- Mean local cortical bone adaptation responses are quasi-linear and bone region specific. Low strain bone regions located near the null axis show very little bone adaptation response.
- High strain compressive and tensile bone regions show similar responses to changes in peak load. However, bone formation in the compressive regions of high strain commence at approximately 2 N lower loading magnitude.

The developed mechanostat model can be used to compare the adaptation response across different mouse tibia axial loading studies that each consider differing peak loads. Additionally, the simulated bone loss due to sciatic neurectomy could be replaced with alternate disease states (e.g. osteoporosis induced through ovariectomy). The numerically efficient simulation framework based on beam theory also provides a means to replace current, mechanical only based models with mechanobiological models of bone adaptation that account for bone cellular interactions.

### Supplementary Information

Below is the link to the electronic supplementary material.

Supplementary file1 (png 332 KB)

## Data Availability

Data used in this study is owned by the University of Bristol. Requests to access the datasets should be direct to PP (peter.pivonka@qut.edu.au).

## Footnotes

1 Note that in endpoint imaging studies, the initial state of the tibia is not generally imaged and comparisons are made between loaded and contralateral tibia at $t_{end}$. However, for bone adaptation simulations, an initial state of the tibia is required to simulate adaptation due to applied loading.
