# Supplementary figures and images for: Cortical bone adaptation response is region specific, but not peak load dependent: insights from μCT image analysis and mechanostat simulations of the mouse tibia loading model
Source: Biomech Model Mechanobiol. 2023 Oct 18;23(1):287–304. doi: 10.1007/s10237-023-01775-6 (PMC10901956; doi:10.1007/s10237-023-01775-6)

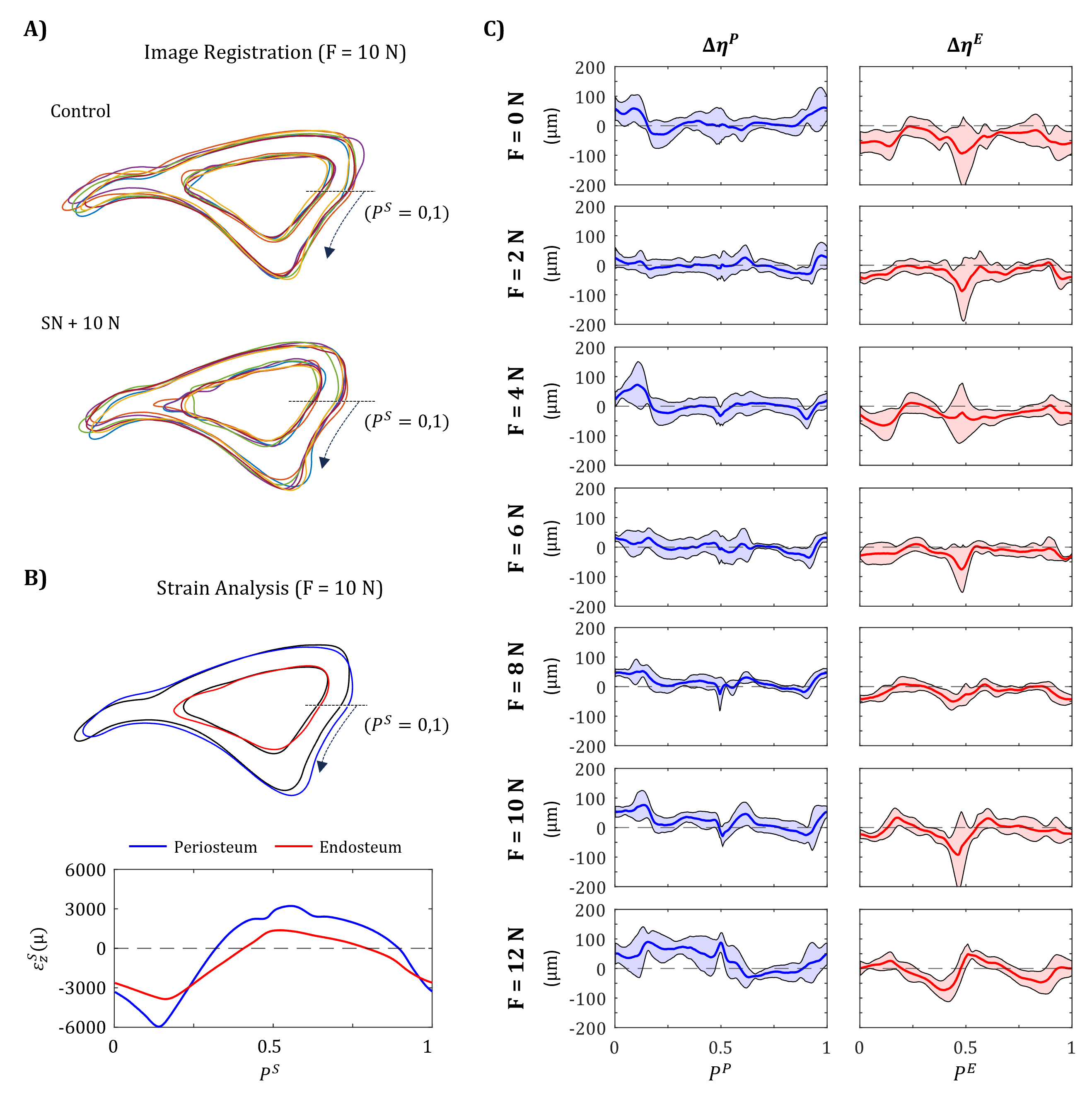

Supplement: Supplementary file 1 — Supplementary file1 (png 332 KB) [file 10237_2023_1775_MOESM1_ESM.png]
